# Case Report: Transrectal contrast-enhanced ultrasonography on preoperative evaluation of rectal neuroendocrine tumors: a 17-case preliminary study

**DOI:** 10.3389/fsurg.2026.1801458

**Published:** 2026-04-28

**Authors:** Jigang Jing, Yuting Wu, Hua Zhuang

**Affiliations:** Department of Ultrasound Medicine, West China Hospital of Sichuan University, Chengdu, Sichuan, China

**Keywords:** contrast-enhanced ultrasonography, enteroscopic ultrasound, neuroendocrine neoplasms, rectal tumor staging, transrectal ultrasound

## Abstract

**Objective:**

To evaluate the feasibility of transrectal ultrasound (TRUS) in the sonographic characterization and preoperative assessment of rectal neuroendocrine neoplasms (NENs).

**Methods:**

Retrospective analysis was performed on the transrectal ultrasound manifestations and clinical data of 17 patients with pathologically and immunohistochemically confirmed rectal NENs [8 cases of rectal neuroendocrine tumors [NETs, G1/G2] and 9 cases of rectal neuroendocrine carcinomas [NECs, G3]] between June 2020 and June 2025. These TRUS findings were compared with the ultrasound features of 31 contemporaneous cases of middle- and lower-segment rectal cancer.

**Results:**

All 8 rectal NETs (G1, G2) showed hypoechoic masses in the rectal mucosal and submucosal layers on TRUS, with clear borders in 8 cases, point-stripe blood flow signals in 7 cases, and round-shaped lymph nodes with a short diameter > 5 mm in 3 cases; contrast-enhanced ultrasound (CEUS) was performed in 6 cases, showing inhomogeneous hyperenhancement in 2 cases and homogeneous isoenhancement in 4 cases. Among the 9 rectal NECs (G3), 8 presented with localized irregular thickening of the rectal wall, 7 invaded perirectal tissues or organs, all lesions were hypoechoic or heteroechoic with muscularis propria infiltration, and 4 cases had enlarged local lymph nodes. Compared with rectal cancer, the distribution of T stage (T1 vs. T2–4) differed significantly (*P* = 0.016), whereas no significant differences were noted in gender, age, distance from the lower margin of the lesion to the intersphincteric sulcus, lesion length, lesion thickness, or N stage (all *P* > 0.05). The overall concordance rate between ultrasonographic staging and surgical pathological staging was 70.6% (12/17) for all rectal NENs, with 87.5% (7/8) for NETs and 55.6% (5/9) for NECs. A limitation of this retrospective feasibility study is its relatively small sample size (*n* = 17), which needs further verification in future multi-center large-sample studies.

**Conclusions:**

Rectal NETs exhibit characteristic TRUS manifestations and require differentiation from polyps, adenocarcinomas, and inflammatory lesions. Transrectal contrast-enhanced ultrasonography (TR-CEUS), though not widely adopted, is feasible for the preoperative assessment of rectal NENs and may be valuable for follow-up after endoscopic resection to monitor recurrence.

## 1 Introduction

Rectal neuroendocrine tumors (NETs) are rare rectal neoplasms that, like rectal malignant melanoma, require differentiation from common rectal cancers (1). With the growing utilization of screening colonoscopies for patients presenting with gastrointestinal symptoms and routine colonoscopies for individuals aged >40 years, the detection rate of rectal NETs has increased steadily (2). Endoscopic ultrasound (EUS) is indispensable in the diagnosis and preoperative evaluation of rectal NETs, facilitating the assessment of residual tumor burden and tumor characteristics to guide clinical management (3). Currently, EUS is generally recommended for lesions >10 mm or those with atypical features (e.g., central depression) (4), and all lesions >5 mm should undergo EUS to rule out muscularis propria invasion prior to resection (5).

However, transrectal ultrasound (TRUS) has recently gained recognition for its advantages in the preoperative evaluation of rectal tumors. Rectal NETs are predominantly located in the middle and lower rectum (2–10 cm from the anus) (6), which falls within the imaging range of TRUS (12–15 cm from the anus), rendering TRUS increasingly applicable for the assessment of rectal NETs. Compared with EUS, TRUS is more accessible, requires no anesthesia, is easy to perform, less time-consuming, and more cost-effective. Given that NETs are smaller than neuroendocrine carcinomas (NECs), TRUS-based screening and evaluation of rectal NETs merit further exploration. Existing literature primarily focuses on the utility of EUS in the evaluation of rectal NETs, with limited data available on TRUS and even fewer studies investigating transrectal contrast-enhanced ultrasound (CEUS) (7, 8). While EUS is well-established for the staging of rectal NETs, its availability is limited in resource-constrained settings. To our knowledge, no previous studies have systematically evaluated TRUS/CEUS—a low-cost, accessible imaging modality—for the staging of rectal NETs. This research gap highlights the novelty of our work: exploring TRUS/CEUS as a potential complementary tool for the assessment of rectal NETs. Given the rarity of rectal NETs, we conducted this retrospective single-center study to investigate the feasibility of TRUS/CEUS, acknowledging the inherent limitations of small sample sizes in rare disease research.

The aim of this study was to evaluate the correlation between TRUS/CEUS staging and surgical pathology in patients with rectal neuroendocrine neoplasms (NENs). Our hypothesis was that TRUS/CEUS would demonstrate preliminary concordance with pathological findings, supporting its potential as a feasible staging tool in resource-limited settings.

## 2 Methods

### 2.1 Study aims and endpoints

#### 2.1.1 Primary aim

To evaluate the feasibility and diagnostic value of transrectal ultrasound (TRUS) in the sonographic characterization and preoperative staging assessment of rectal neuroendocrine neoplasms (NENs).

#### 2.1.2 Secondary aims

(1) To analyze the differences in TRUS manifestations between rectal NENs (including G1/G2 NETs and G3 NECs) and middle- and lower-segment rectal cancer;
(2) To explore the concordance rate between TRUS staging and surgical pathological staging in rectal NENs; (3) To provide imaging evidence for the differential diagnosis and preoperative evaluation of rectal NENs.

#### 2.1.3 Primary endpoint

The concordance rate between TRUS-based preoperative staging and surgical pathological staging of rectal NENs, which was used to evaluate the feasibility of TRUS in preoperative assessment.

#### 2.1.4 Secondary endpoints

(1) The sensitivity and specificity of TRUS in identifying the sonographic characteristics of rectal NENs (such as lesion location, size, echo, border, blood flow signals); (2) The differences in TRUS-related indicators (lesion length, thickness, T/N staging) between rectal NENs and rectal cancer; (3) The detection rate of TRUS for perienteric lymph node enlargement and distant metastasis in rectal NENs patients.

### 2.2 Study subjects

#### 2.2.1 Patient selection criteria and process

The patient population was selected based on strict inclusion and exclusion criteria between June 2020 and June 2025, which were formulated in accordance with the study aims and international relevant guidelines.

Inclusion criteria were as follows: (1) Patients who were pathologically and immunohistochemically confirmed to have rectal neuroendocrine neoplasms (NENs, including G1/G2 NETs and G3 NECs) or middle- and lower-segment rectal adenocarcinoma, with clear pathological diagnosis reports; (2) Patients who underwent transrectal ultrasound (TRUS) examination before any anti-tumor treatment (including surgery, chemotherapy, radiotherapy, etc.), and the ultrasound data were complete and available for analysis; (3) Patients with complete clinical data, including demographic information (gender, age), clinical symptoms, imaging findings (TRUS, CT, MRI, etc.), pathological results (pathological type, grade, staging), and follow-up data (if available); (4) Patients who signed the written informed consent form voluntarily and agreed to participate in the study.

Exclusion criteria were: (1) Patients with incomplete clinical data, ultrasound data or pathological data, or lost to follow-up during the study period; (2) Patients who had received anti-tumor treatment (such as chemotherapy, radiotherapy, targeted therapy, immunotherapy) before TRUS examination, which may affect the ultrasound manifestations of lesions; (3) Patients with other malignant tumors (except the studied rectal lesions) or severe systemic diseases (such as severe heart, liver, kidney diseases, coagulation disorders), which may affect the study results or patient tolerance; (4) Patients who refused to sign the informed consent form or withdrew from the study voluntarily; (5) Patients with rectal lesions combined with other benign diseases (such as severe rectal inflammation, rectal polyps larger than 10 mm) that may interfere with the sonographic characterization of NENs.

The selection process was carried out by two independent researchers: first, screening patients who met the inclusion criteria from the electronic medical record system of our hospital; then, excluding patients who met the exclusion criteria; finally, reviewing and confirming the eligible patients through joint discussion to ensure the accuracy of the study subjects. Disagreements between the two researchers were resolved through consultation with a third senior researcher.

#### 2.2.2 Rectal neuroendocrine tumor (NET) patients (G1, G2)

Based on the above selection criteria, 8 patients with pathologically and immunohistochemically confirmed rectal NETs (G1, G2) were enrolled. All patients were positive for synaptophysin (Syn) and Ki-67. There were 4 males and 4 females, with an age range of 37–81 years (mean: 50.50 ± 15.21 years). Colonoscopy detected rectal submucosal masses in 5 cases, 2 cases presented with hematochezia, and 1 case suffered from recurrent lower abdominal pain. Among these patients, 5 were classified as G1 and 3 as G2.

#### 2.2.3 Rectal cancer patients

According to the same selection criteria, 31 patients with pathologically and immunohistochemically confirmed middle- and lower-segment rectal cancer [including 9 G3 neuroendocrine carcinoma (NEC) cases] were recruited as controls. There were 18 males and 13 females, aged 35–92 years (mean: 59.48 ± 12.96 years). All patients underwent preoperative ultrasound staging after hospitalization. Among them, 5 patients underwent thoracoabdominal computed tomography (CT), with 4 excluding distant metastasis and 1 identifying distant metastasis; 1 patient underwent positron emission tomography-computed tomography (PET-CT), which confirmed metastasis; and 4 patients underwent preoperative rectal magnetic resonance imaging (MRI). Rectal NETs differ biologically and clinically from adenocarcinomas; however, sonographic diagnosis of rectal NETs requires differentiation from adenocarcinomas and other lesions prior to staging.

#### 2.2.4 Rectal neuroendocrine carcinoma (NEC) patients (G3)

Among the enrolled patients, 9 were pathologically and immunohistochemically confirmed to have rectal NECs (G3), all of whom were positive for Syn and Ki-67 and met the aforementioned selection criteria.

### 2.3 Ethical approval

This study was approved by the Biomedical Ethics Review Committee of West China Hospital of Sichuan University [approval number: 1789 (2025)].

## 3 Instruments and methods

### 3.1 Instruments

An Esaote My Lab Twice ultrasonic diagnostic system (Esaote, Genoa, Italy) equipped with a biplane transrectal probe (TRT33; linear frequency: 4–13 MHz; convex frequency: 3–9 MHz) was used for real-time contrast-enhanced ultrasound (CEUS) and preoperative transrectal ultrasound (TRUS).

A Mindray A20 ultrasonic diagnostic system (Shenzhen Mindray Bio-Medical Electronics Co., Ltd., Shenzhen, Guangdong Province, China) equipped with a biplane transrectal probe (ELB4-13; linear frequency: 4–13 MHz; convex frequency: 3–10 MHz) was employed for preoperative TRUS and real-time CEUS. In cases of luminal stenosis, a transvaginal probe (CV2-10U; convex frequency: 2–10 MHz) was used for endorectal exploration to enable end-fire scanning. A volumetric probe(D4-8;frequency4-8 MHz) or convex abdominal probe（C1-5;frequency1-5 MHz was selected when necessary.

### 3.2 Examination methods

#### 3.2.1 Pre-examination preparation

Prior to the examination, all patients underwent bowel preparation via rectal enema with 40 mL of glycerol, administered one hour before the procedure.

#### 3.2.2 Examination procedure

Patients were placed in the left lateral decubitus position with flexed hips and knees. The anus was fully exposed by the patient abducting their buttocks with the right hand. The probe surface was coated with coupling agent, covered with a latex sheath (with air completely expelled), and an additional layer of coupling agent was applied externally. All examinations were performed by a sonographer with more than 5 years of TRUS experience using an intracavitary biplane probe. Dynamic grayscale images of the rectal tumor were acquired in both convex and linear array modes. Tumor location was marked using clock face notation (with the patient in the lithotomy position), and the lowest point of the tumor was independently verified in both modes. Instrument parameters were optimized according to individual patient conditions; in cases of luminal stenosis, a transvaginal probe was used for intrarectal examination.Based on colonoscopy or digital rectal examination findings, for male patients with rectal luminal stenosis: >8 cm from the anal verge, transvaginal/end-fire transducers are used endorectally after informed consent; <8 cm perineal volumetric transducers with 3D/4D imaging to assess lesion-wall relationship and adjacent organ involvement; 10–15 cm, left lower abdominal convex transducers, preferably with a full bladder.

#### 3.2.3 Observation indicators

The following indicators were recorded: lesion length, lesion thickness, distance from the lower margin of the lesion to the intersphincteric sulcus, and ultrasonic T/N staging. Tumor size was measured in two orthogonal planes by the same operator, and the mean values were used for statistical analysis.

#### 3.2.4 Staging criteria

NET T-staging was performed in accordance with the 2023 European Neuroendocrine Tumour Society (ENETS) guidelines for gastrointestinal NETs (9). Rectal cancer ultrasound staging followed the criteria of the Union for International Cancer Control (UICC) (10). N staging was defined as positive if lymph nodes had a short diameter ≥5 mm, a round shape, and an indistinct cortex-medulla boundary; N staging was negative if lymph nodes had a short diameter <5 mm, a flat shape, a distinct cortex-medulla boundary, or were undetectable (11).

#### 3.2.5 CEUS procedure

After clear visualization of the lesion, 2.4 mL of Sonovue was injected intravenously, and CEUS mode was activated immediately with 120 s of image storage. Enhancement patterns of the lesion and perilesional tissues were observed, and T/N staging was supplemented or adjusted accordingly based on the CEUS findings.

#### 3.2.6 Quality control

**Figure 1:**
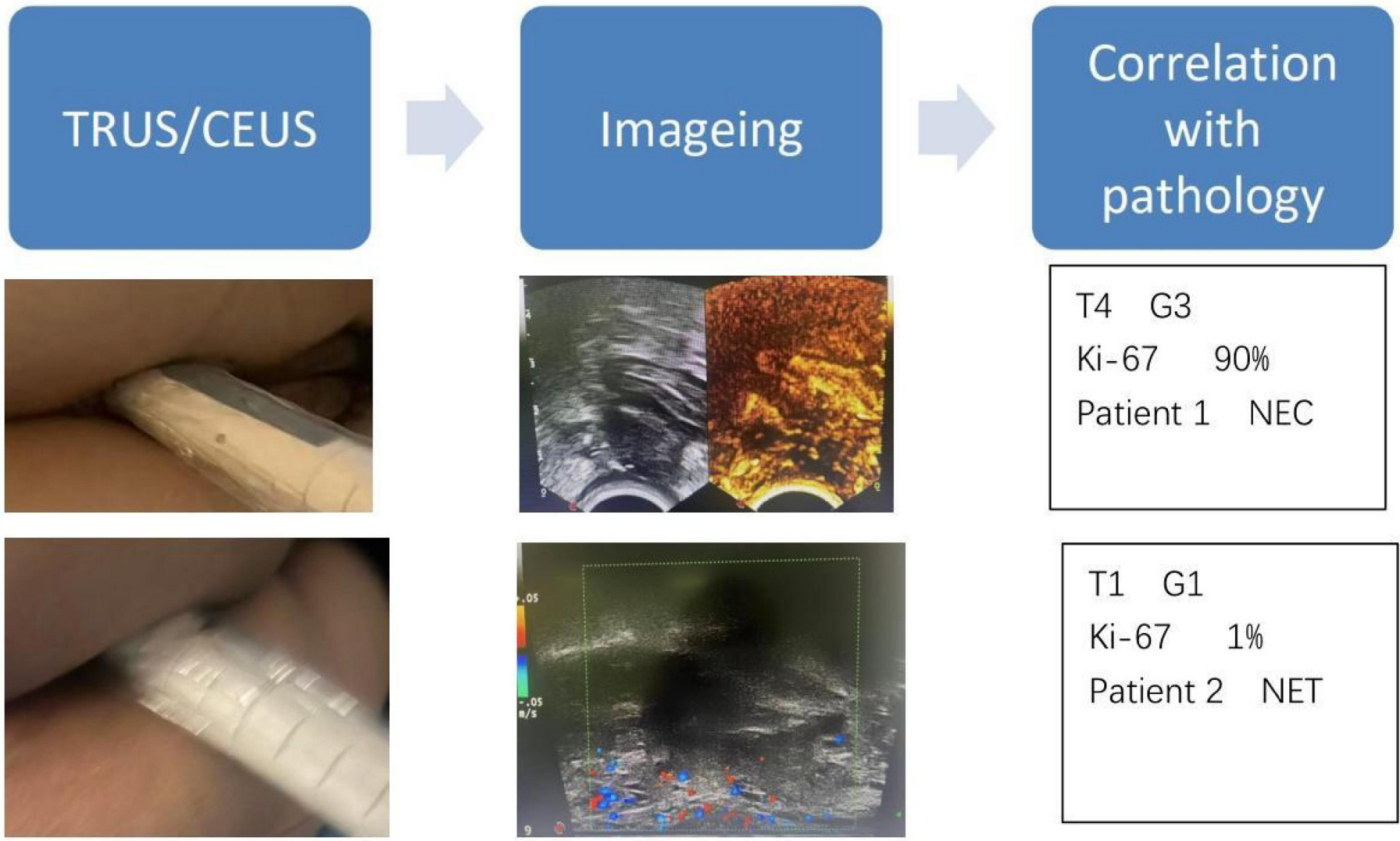
Schematic summary of TRUS/CEUS workflow and correlation with pathology.

All TRUS and CEUS examinations were performed by two board-certified abdominal sonographers with more than 5 years of experience in rectal imaging. To minimize interpretation bias, the sonographers were blinded to the final surgical pathology results and prior colonoscopy/imaging findings (e.g., MRI) during image acquisition and staging. For the assessment of interobserver reproducibility, both sonographers independently reviewed the stored images of all cases (blinded to each other's interpretations) to determine tumor size, invasion depth, and staging. Any discrepancies were resolved through consensus with a third senior radiologist (with more than 10 years of experience), who was also blinded to the pathology results. Intraclass correlation coefficients (ICCs) were calculated: ICC was 0.88 for tumor size and 0.86 for invasion depth, indicating excellent interobserver agreement (12). A schematic diagram of the TRUS/CEUS workflow and its correlation with pathology is shown in Figure 1.

## 4 Statistical analysis

Statistical analysis was performed using SPSS 27.0 software (IBM Corp., Armonk, NY, USA). Continuous data were compared using independent samples t-tests, and categorical data were analyzed using Fisher's exact test. A *P*-value <0.05 was considered statistically significant.

## 5 Results

**Figure 2:**
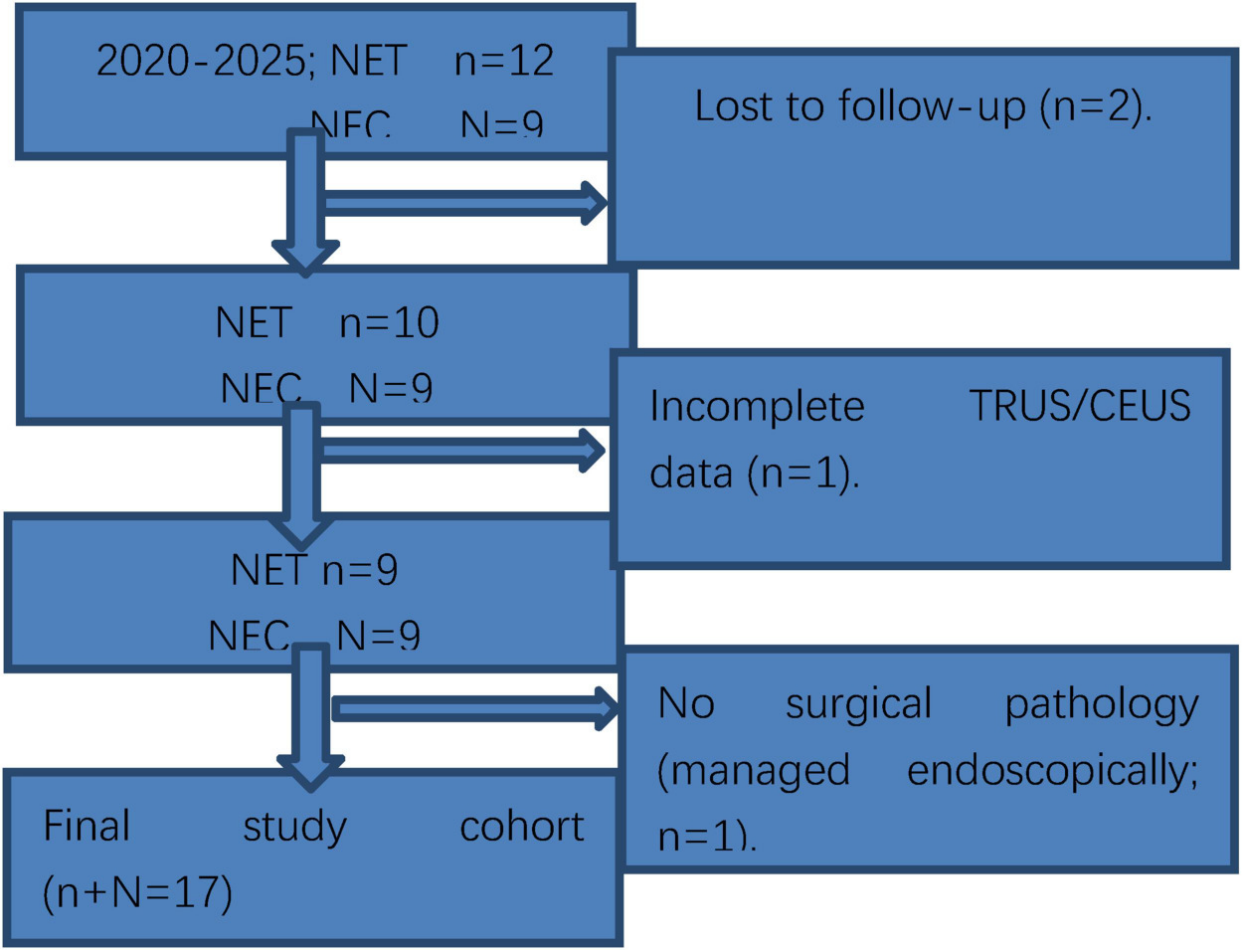
Patient flow diagram.

The patient selection process is detailed in Figure 2 (Patient Flow Diagram), which clearly shows the screening, inclusion and exclusion process of all study subjects, ensuring the transparency and rationality of patient enrollment.

### 5.1 Rectal NETs (grades G1 and G2)

**Figure 3:**
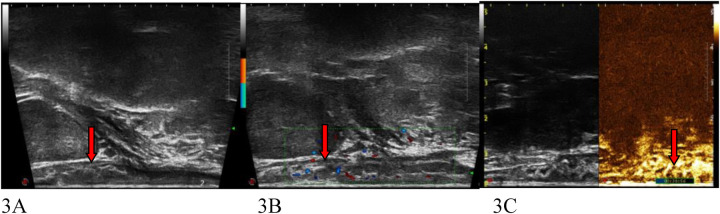
Patient 1,M,35Y. A submucosal lesion 5 cm from the anal verge was revealed by endoscopy. While TRUS image of a rectal NET, in size of 8 mm hypoechoic lesion (arrow) is visualized 5 cm from the anal verge, pathology confirmed submucosal invasion [T1], surgical pathology later confirmed submucosal invasion, consistent with TRUS findings. **(A)** 8 mm hypoechoic NET. **(B)** Blood flow signals within NET. **(C)** Isoechoic enhancement in CEUS.

Transrectal ultrasound (TRUS) demonstrated hypoechoic mucosal or submucosal masses in all 8 NET cases (Figure 3), 3 of which involved the muscularis propria. Lesion sizes ranged from 8 × 4 × 6 mm to 42 × 18 × 16 mm. Five cases presented with clear borders, 4 with regular morphology, and 6 with homogeneous internal echogenicity. Punctate calcification was observed in 1 case, and patchy anechoicity in another. Punctate blood flow signals were detected in 7 cases, arterial spectra were identifiable in 4 cases, and perienteric lymph nodes with a short diameter >5 mm were noted in 3 cases. Contrast-enhanced ultrasound (CEUS) was performed in 6 cases, revealing inhomogeneous hyperenhancement in 2 cases and homogeneous isoenhancement in 4 cases.

The concordance rate between ultrasonic staging and surgical pathology was 87.5% (7/8) for NETs. When compared with 31 cases of rectal cancer, NETs exhibited a significant difference in T staging distribution (T1 vs. T2–4, *P* = 0.016); however, no significant differences were observed in gender, age, distance from the lower lesion margin to the intersphincteric sulcus, lesion length, lesion thickness, or N staging (all *P* > 0.05) (Table 1).

**Table 1:** Comparison of clinical and ultrasonographic features between 8 cases of rectal neuroendocrine tumors and 31 low and intermediate rectal carcinomas .

Gender and ultrasound characteristics	Rectal neuroendocrine tumors (*n* = 8)	Low and intermediate rectal carcinomas (*n* = 31)	*P*-value
Gendar			
M	4	18	
F	4	13	0.709
Age	50.50 ± 15.21	59.48 ± 12.96	0.100
distance from the lower margin to the intersphincteric sulcus	38.75 ± 20.67	45.03 ± 24.25	0.507
Length of lesion	25.25 ± 10.57	31.06 ± 9.52	0.140
Thickness of lesion	9.75 ± 7.15	11.42 ± 4.28	0.544
Tstaging			
T1	5	5	
T2∼4	3	26	0.016
Nstaging			
N+	3	11	
N-	5	20	1.000

N + lymph nodes had a short diameter ≥5 mm, round shape, and indistinct cortex-medulla boundary.

N-lymph nodes had a short diameter <5 mm, flat shape, distinct cortex-medulla boundary, or were undetectable.

Treatments for NETs were as follows: 1 case underwent endoscopic submucosal dissection (ESD), 1 case underwent endoscopic mucosal resection (EMR), 1 case underwent transanal rectal tumor resection, 4 cases underwent laparoscopic radical low anterior resection, and 1 case received conservative medical treatment.

Measurements of the maximum tumor diameter were consistent between ultrasound and colonoscopy in 3 NET cases, inconsistent in 3 cases (with ultrasound measurements being larger), and not comparable in 2 cases due to the absence of colonoscopic measurement data.

### 5.2 Rectal NECs (grade G3)

Among the 9 NEC cases, 8 presented with localized irregular thickening of the rectal wall, 7 invaded perirectal tissues or organs, and all lesions were hypoechoic or heteroechoic with muscularis propria involvement. Local lymphadenopathy was observed in 4 cases. The concordance rate between ultrasonic staging and surgical pathology was 55.6% (5/9) for NECs.

For all 17 rectal NENs, the overall concordance rate between ultrasonic staging and surgical pathology was 70.6% (12/17). Pathological features of NETs and NECs are compared in Table 2.

**Table 2:** Comparison of pathological changes between 8 cases of NET and 9 cases of NEC in this group.

Variable	NET (*n* = 8)	NEC (*n* = 9)
Tumor grade G		
G1	5	0
G2	3	0
G3	0	9
Ki-67	4.56% ± 5.53%	54.44% ± 21.86%
Distant metastasis	1	3

## 6 Discussion

Neuroendocrine neoplasms (NENs) were historically referred to as “carcinoids,” first described by Langhans in 1867. In 1914, Gosset and Mason observed that although carcinoids share histological features with carcinomas, their biological behavior is distinct, characterized by slow progression and endocrine-related properties (13). The incidence of gastroenteropancreatic (GEP) NETs is steadily increasing in North America, Asia, and Europe, with the most pronounced rise in North America. Regional variations in GEP NET distribution exist: small intestinal and rectal NETs are most prevalent in North America, rectal and pancreatic NETs in Asia, and small intestinal and pancreatic NETs in Europe (14). The rectum is the second most common site for NETs (after the small intestine), accounting for 12%–27% of all gastrointestinal NENs and 1%–2% of all rectal tumors (15).

Endoscopic ultrasound (EUS) facilitates the assessment of lesion size, invasion depth, and lymph node metastasis. Recent European Neuroendocrine Tumor Society (ENETS) guidelines do not specify the timing of EUS utilization (9), but EUS may be unnecessary for rectal NETs <10 mm in size (16). In contrast to EUS, transrectal ultrasound (TRUS) inserts the probe directly into the rectum to provide high-resolution imaging of the rectum, anal canal, and surrounding organs, rendering it suitable for evaluating tumor invasion depth and internal orifices of anal fistulas. The procedure is brief (lasting several minutes) and well-tolerated. Transrectal contrast-enhanced ultrasound (CEUS) further clarifies the blood supply of rectal and anal canal lesions. In our NET cohort, TRUS demonstrated mucosal/submucosal hypoechoic masses with variable border clarity, morphology, and internal echogenicity—consistent with typical EUS findings of rectal NENs [hypoechoic, homogeneous submucosal lesions (17)]. CEUS revealed homogeneous isoenhancement in most NET cases, which may be associated with small lesion size and predominant tumor cell components, whereas inhomogeneous enhancement in neuroendocrine carcinomas (NECs) may be related to larger lesion size, lesion necrosis, and post-treatment changes.

It is worth noting that positron emission tomography-computed tomography (PET-CT) plays an irreplaceable role in the comprehensive evaluation of rectal NETs, especially in the assessment of distant metastasis and tumor grading, which is crucial for formulating individualized treatment strategies. Previous studies have confirmed that PET-CT, based on its ability to detect abnormal glucose metabolism, can effectively identify primary lesions, regional lymph node involvement, and distant metastases of NETs, thereby improving the accuracy of clinical staging and avoiding missed diagnosis of occult metastases. A study (18) emphasized that PET-CT has high sensitivity and specificity in the staging of mid-to-high grade NETs, and can provide important reference for judging tumor aggressiveness and prognosis. In our study, 1 patient with rectal cancer (including NEC cases) underwent PET-CT, which confirmed distant metastasis, further indicating the value of PET-CT in identifying distant spread of neuroendocrine neoplasms. For rectal NETs with suspected distant metastasis or unclear staging, PET-CT can complement the limitations of TRUS in detecting distant lesions, forming a multi-modal imaging evaluation system together with TRUS, EUS and other modalities to improve the comprehensiveness and accuracy of preoperative evaluation.

Tumor size measurements differed between TRUS and colonoscopy in some cases, likely because TRUS better visualizes the entire tumor base. Endoscopic size determination is reported to have low accuracy (19), yet tumor size directly guides clinical management (20)—traditionally based on endoscopic measurements. Our findings suggest that TRUS measurements may be closer to the true tumor size. The mean maximum lesion diameter in our NET cohort was 25 mm, larger than the previously reported 6.8 mm (range: 2.3–13.7 mm) (6). This discrepancy may arise from the low detection rate of small NETs by TRUS (a key limitation of our study) and selection bias: smaller submucosal lesions (<10 mm) detected by colonoscopy are often resected directly without imaging, leaving larger lesions (>10 mm) for EUS/TRUS assessment.

Potential false negatives in TRUS/CEUS, particularly for small rectal NETs (<10 mm), warrant attention. Since all lesions in our cohort were ≥10 mm, we could not evaluate the modality's ability to detect smaller tumors. Small NETs may be missed due to their size or overlap with normal rectal wall echoes, whereas colonoscopy (with direct mucosal visualization) is more sensitive for detecting these lesions. Thus, TRUS/CEUS is best used as a complementary tool: colonoscopy identifies small lesions and enables biopsy, while TRUS/CEUS provides staging information (invasion depth) in resource-limited settings lacking EUS. For lesions ≥10 mm (where staging is critical for treatment planning), TRUS/CEUS may offer a pragmatic alternative, though EUS remains the gold standard in high-resource centers.

The detection rate of small rectal NETs can be improved by rectal aqueous or coupling agent filling followed by TRUS or transrectal dual CEUS. Additionally, transvaginal probes can be used for transrectal examination in both genders (21). Established evidence indicates that transvaginal probes overcome the difficulty of detecting proximal tumors with biplane transrectal probes in cases of luminal stenosis. In female patients, adding a transvaginal scanning pathway provides additional information, improves T/N staging accuracy (22–24), identifies extramural vascular invasion (EMVI) (25–27) and circumferential resection margin (CRM) (28–32), and facilitates multimodal imaging (e.g., ultrasound elastography, dual CEUS). For all 17 rectal NENs, the overall concordance rate between ultrasonic staging and surgical pathology was 70.6% (12/17). The distribution of case numbers in T1 and T2∼4 stages differed significantly between 8 rectal NET cases and 31 low- and intermediate-grade rectal cancer cases (*P* = 0.016), which may be attributed to the lower invasiveness of rectal NETs compared with rectal adenocarcinoma and NEC. However, no significant differences were observed in gender, age, distance from the lower margin to the intersphincteric sulcus, lesion length, lesion thickness, or N stage (*P* > 0.05), indicating that clinical diagnosis of NENs still requires pathological examination and immunohistochemical analysis. This suggests that the procedure and method of endoscopic ultrasound staging for rectal cancer are also applicable to rectal NENs in most cases.

### 6.1 Novelty of the study

This study is among the first to investigate TRUS/CEUS for the staging of rectal NETs. Previous research has focused on EUS, magnetic resonance imaging (MRI), or colonoscopy, but TRUS/CEUS offers unique advantages (e.g., portability, lack of ionizing radiation, lower cost) that could address unmet needs in settings without EUS access. Our preliminary data on the correlation between TRUS/CEUS and surgical pathology (70.6% concordance) provides a novel foundation for future validation studies.

### 6.2 Strengths

This study has several strengths. First, it focuses on a rare disease (rectal NETs) where data on alternative staging modalities are scarce, addressing an unmet research gap. Second, we incorporated blinding of sonographers to pathology results and assessed interobserver reproducibility, minimizing bias and enhancing methodological rigor. Third, we correlated TRUS/CEUS findings with surgical pathology (the reference standard), ensuring valid assessment of staging accuracy. Finally, the study highlights the potential of TRUS/CEUS—an accessible, low-cost modality—for settings with limited EUS access, addressing a pragmatic clinical need.

### 6.3 Limitations

As noted earlier, the primary limitations of this study include its retrospective design and small sample size (*n* = 17), the potential impact of selection bias and information bias on the research results, which limit statistical power and generalizability. Additional limitations include the single-center design (potential institutional bias) and the exclusion of small lesions (<10 mm), precluding assessment of TRUS/CEUS for detecting early-stage NETs. Third, we did not directly compare TRUS/CEUS with EUS (the gold standard), so we cannot comment on its equivalence to EUS. Future studies conducting head-to-head comparisons of TRUS/CEUS vs. EUS in the same patient cohort to validate diagnostic accuracy are highly necessary.Finally, focusing on establishing the ultrasound strategy, only 4 out of 17 cases of neuroendocrine tumors in this group underwent MRI examination,this study did not conduct a comparative analysis of TRUS and MRI.

### 6.4 Future directions

Future research should address the limitations of this feasibility study. First, prospective, multicenter studies with larger sample sizes (including lesions <10 mm) are needed to validate TRUS/CEUS staging accuracy and calculate robust diagnostic metrics (sensitivity, specificity). Second, head-to-head comparisons of TRUS/CEUS vs. EUS are essential to determine whether TRUS/CEUS can serve as a true alternative to EUS. Third, cost-effectiveness analyses would help contextualize the value of TRUS/CEUS in resource-constrained settings. Forth, it is importent comparing TRUS and MRI and calculating their concordance in the follow-up prospective study.Finally, long-term follow-up studies could assess the role of TRUS/CEUS in post-resection surveillance for recurrence.

### 6.5 Potential role of TRUS/CEUS in clinical practice

TRUS/CEUS may serve as a triage tool for small rectal NETs (<10 mm), where invasive staging with EUS may be unnecessary. It may be a more accessible, cost-effective, and repeatable alternative in centers without EUS. It could also be useful for follow-up after endoscopic resection to monitor for recurrence. Importantly, TRUS/CEUS is not a replacement for EUS/MRI in high-risk cases (e.g., large tumors, high-grade NETs), and its role is complementary to existing standards.

Rectal NENs exhibit characteristic TRUS findings and require differentiation from polyps, adenocarcinomas, and inflammatory lesions. Although not widely adopted, transrectal CEUS is feasible for the preoperative evaluation of rectal NENs and may be useful for post-endoscopic resection follow-up to monitor recurrence. TRUS/CEUS offers unique advantages (e.g., portability, lack of ionizing radiation, lower cost) that could address unmet needs in settings without EUS access.

## Data Availability

The original contributions presented in the study are included in the article/Supplementary Material, further inquiries can be directed to the corresponding author.
